# Decreased risk-proneness with increasing age in equally raised and kept wolves and dogs

**DOI:** 10.1371/journal.pone.0313916

**Published:** 2025-01-08

**Authors:** Hillary Jean-Joseph, Kim Kortekaas, Friederike Range, Kurt Kotrschal

**Affiliations:** 1 Department of Behavioural and Cognitive Biology, University of Vienna, Vienna, Austria; 2 Domestication Lab, University of Veterinary Medicine, Vienna, Medical University of Vienna, University of Vienna, Vienna, Austria; Indian Institute of Science Education and Research Kolkata, INDIA

## Abstract

A basic mechanism of domestication is the selection for fearlessness and acceptance of humans as social partners, which may affect risk-taking behavior and the ability to use humans as social support, both at the behavioural and physiological levels. We combined behavioural observations with heart rate parameters (i.e., HR and heart rate variability, HRV) in equally raised and housed wolves and dogs to assess the responses to food offered in the vicinity of a potential stressor (an unknown spinning object) with and without social support from a familiar human. Based on previous studies on neophobia in wolves and dogs, we expected dogs to be less scared of the object, approach more quickly, show less ambivalent behaviour, lower HR, and higher HRV, than wolves, especially at the presence of a human partner. However, we found that mainly age and the presence of a familiar human affected the behaviour of our subjects: older wolves and dogs were generally bolder and faster to approach the food and the familiar human’s presence increased the likelihood of taking it. HR rate parameters were affected by age and the stage of the test. Wolves and dogs showed particularly high HRs at the beginning and end of the test sessions. We conclude that in our paradigm, wolves’ and dogs’ risk-proneness varied with age, rather than species. Additionally, the presence of a familiar human increased the motivation of both, dogs and wolves to take the food.

## Introduction

Engaging in potentially dangerous activities may raise fear and stress, affecting behavior and physiology by activating the autonomic nervous system (ANS) and the hypothalamo-pituary-adrenal axis [1], triggering “flight”, “fight” or “freeze” behaviors [2]. Physiological parameters affected include cardiac rhythmicity [3–8], such as heart rate (HR) and heart rate variability (HRV), making them relevant indicators of both the physiological and affective states of a subject [9–12]. In fact, HR was previously used to measure the response of dogs to different types of stimuli, including those which would potentially elicit fear responses [13, 14]. Risk-taking, defined as any controlled behaviour with a perceived uncertainty about its outcomes [15], can trigger such physiological responses. By presenting food in a situation that may potentially be considered dangerous, risk-taking behaviours and (potentially) associated physiological responses can be observed, with risk-proneness being the propensity to be attracted to, or the willingness to tolerate, options that entail a potentially high risk of loss (according to the APA Dictionary of Psychology).

Dogs, particularly in comparison to wolves, are an ideal species to study how domestication has affected risk-taking behaviour and risk-proneness [16, 17]. Reduced fear and stress responsiveness are common components of the domestication syndrome present in most, if not all, domesticated animals [18–20]. As dogs are the domesticated form of wolves, they should be less neophobic, i.e., less unwilling to engage in novel situations and stimuli [21]. Hence, dogs should show a greater risk-proneness, i.e. perceive a certain situation as less risky than wolves with the same lifetime experiences. Dogs have indeed, been found to be less neophobic than wolves [22, 23], which were, however, more explorative and persistent in interacting with novel objects than dogs [24, 25]. In contrast, wolves previously turned out to be more risk-prone than dogs. When presented with 100% odds of obtaining a piece of kibble or with 50% odds for either a piece of meat or nothing, wolves, but not dogs, chose the risky option [26].

Different feeding ecologies could explain differences in risk-proneness [27]: whereas wolves are mainly cooperative hunters [28, 29], free-ranging dogs tend to scavenge on human waste [30, 31]. In fact, dogs are adapted to an agriculturalist human diet as they are better at digesting starch than wolves [32]. Accordingly, wolves, due to their lifestyle, should be more neophobic than dogs but also more persistent and risk-prone. These predictions are in line with studies showing that species or populations dealing with unreliable food sources are more risk-prone than those living on a relatively stable food supply (tits [33], apes [34, 35]).

Personality is a factor that might influence risk-taking behaviour at the individual level. Indeed, the bold-shy personality dimension has been found from invertebrates [36] to mammals [37, 38], including dogs [39, 40] and wolves [41]. Boldness includes the readiness to deal with uncertainty and take risks [42, 43]. As such, bold individuals are more risk-prone than shy ones. Boldness may vary with age as, for example, older female seals [44] or female eiders [45] were found to be bolder than younger ones. In contrast, boldness in dogs seems to decrease with increasing age [46–48], whereas older and more experienced wolves seem to be bolder [49, 50].

Finally, individual risk-proneness may also be affected by social environment [51, 52]. In fact, both dogs and wolves were more risk-prone when approaching novel objects in the presence of their pack mates [22]. Dogs may be a special case, as the *Hypersociability Hypothesis* suggests they, compared to socialized wolves, not only seek more often social interactions with conspecifics, but also with humans [53]. In fact, dogs benefit from the presence of their caretakers in stressful situations [54–59] and evidently use them as a safe base [56, 60, 61]. Furthermore, it has been proposed that the selection for reduced fearfulness supported the development of certain cognitive skills in dogs compared to wolves, enabling them to use human communicative cues (*Emotional Reactivity Hypothesis*, [62]). It remains unclear whether being able to benefit from humans as social support and motivators in risky or stressful situations is indeed due to domestication or rather an effect of experience with humans [63]. Due to their social organization, wolves need to heavily rely on their group members [28]. Their cooperative social system may enable them to accept humans as social partners and supporters if properly socialized. In line with this, wolves were shown to have all the necessary skills and are motivated to successfully cooperate with humans, including high social tolerance, attentiveness and reading human communicative cues [64–67]. Similar to dogs, they can engage in attachment-like relationships with humans [68–70] and cooperative training sessions with people decreased salivary cortisol in both, dogs and equally raised and kept wolves [71]. In fact, cooperation seems to be at the core of both, wolf social organization and human-dog relationships [63]. Consequently, the *Canine Cooperation Hypothesis* suggests that the dogs’ basic abilities to cooperate and pay attention to humans were not newly acquired during domestication but are derived from the wolves’ capacity to cooperate with their conspecifics [72].

Along similar lines, the *Two Stage Hypothesis* [73] proposes that dogs are highly cooperative with humans because they 1) accept humans as social companions, which is acquired early in life through socialization and 2) learn from humans to follow their gestures and actions through conditioning. For example, puppies improve at following proximal pointing gestures to find hidden food as they get older; thus, their understanding of human communication is not just “innate”, and exposure can ameliorate it [74]. Conversely, wolves do not typically live with humans and thus do not learn to pay attention to humans. Accordingly, if socialized with humans and exposed to many instances where they can learn to pay attention to humans and their actions, the *Two Stage Hypothesis* predicts that both wolves and dogs would be able to benefit from the presence of a human in a stressful situation and pay attention to human motivating gestures and actions.

In the current study, we presented equally socialized and kept dogs and wolves with a risk-taking paradigm in a foraging context. We used cardiac parameters and behaviours to test whether domestication would have affected risk-proneness in dogs as compared to wolves, and to assess whether and to what extent wolves and dogs would use a familiar person as a social support and motivator during the challenge. According to selection for reduced fearfulness as the basic mechanism of domestication, the hypothesis (above) predicts that dogs would show relatively moderate responses to the apparatus, show less fear-related behaviour and a reduced physiological reaction as compared to wolves. Therefore, dogs should be more risk-prone, showing shorter latencies to reach the food and spending more time in its proximity than the wolves. Furthermore, in line with the *Hypersociability Hypothesis* [53] and the *Emotional Reactivity Hypothesis* [75], social support by a familiar human and their communicative actions to engage the subjects with the apparatus would prompt dogs more than human-socialized wolves to take the food. This would also align with our previous results [76, 77].

In contrast, the *Canine Cooperation Hypothesis* [72] and the *Two Stage Hypothesis* [73] would not predict major differences between equally raised and kept dogs and wolves; due to their similar positive exposure to humans during their early development, they regard humans as social partners and pay attention to their motivating gestures. Individual dogs and wolves would overlap in a continuum ranging from fearful to not fearful and risk-prone to risk-avoidant. This would indicate that the main factors affecting the animals during our tests would be life history parameters such as age and/or personality, rather than domestication.

## Methods and materials

### Ethical statement

This research was discussed and approved by the institutional ethics committee at the University of Veterinary Medicine, Vienna, in accordance with Good Scientific Practice guidelines and national legislation (ETK-10/11/2018). All the animals participating in the study were housed at the Wolf Science Center (WSC; www.wolfscience.at), located in the Game Park Ernstbrunn in Austria (License No. AT00012014), and will be kept there under optimal welfare conditions after the end of the study for their entire lifespan. Throughout the study, animals were exposed to a potential stressor–a new object that was rotating and producing noise. However, the exposition happened in a familiar environment and throughout the test, the animals had full agency over their behaviour and were able to choose if they remained in proximity of the stressor or to avoid it. In fact, the animals showed no dramatic stress responses in this experiment, neither in their behaviour, nor physiologically.

### Subjects

Our subjects were wolves, *Canis lupus occidentalis* (N = 13) and dog mongrels, *Canis lupus familiaris* (N = 15) raised and housed in the same way at the Wolf Science Center (WSC) in Ernstbrunn, Austria (see Table 1). All wolves and dogs were born in captivity and hand-raised by humans in a standard way from 10 days of age before being integrated into already existing packs at five months of age [66, 78]. All animals are kept year-round in outside enclosures ranging from 2000 to 8000 m^2^; all enclosures contain bushes, trees, rocks, shelters, and water points providing water *ad libitum* to the animals. The subjects were between 2 and 9 years of age when tested (wolves: median (range) = 6 (2–9); dogs: median (range) = 7 (4–8)) and weighed between 17 and 52 kg (wolves: median (range) = 40 (28–52); dogs: median (range) = 25 (17–34)), see Table 1 for details). The wolves were fed carcasses of deer, rabbit, or chicken three to four times a week while the dogs were fed commercial dog food daily. In addition, dogs were regularly provided with small pieces of deer, rabbit, or chicken to make wolf and dog feeding as similar as possible.

**Table 1:** List of the subjects.

Individual	Species	Sex	Date of birth	Weight in kg*	1^st^ condition
Amarok	Wolf	♂	4.04.2012	40.25	NS
Aragorn	Wolf	♂	4.05.2008	49.20	NS
Chitto	Wolf	♂	7.04.2012	44.57	S
Etu	Wolf	♂	4.05.2016	50.07	S
Geronimo	Wolf	♂	2.05.2009	40.25	S
Nanuk	Wolf	♂	28.04.2009	44.10	NS
Shima	Wolf	♀	4.05.2008	39.70	NS
Taima	Wolf	♀	4.05.2016	27.85	NS
Tala	Wolf	♀	4.04.2012	37.45	S
Tekoa	Wolf	♂	4.05.2016	34.05	NS
Una	Wolf	♀	7.04.2012	32.95	NS
Wamblee	Wolf	♂	22.04.2012	37.25	S
Yukon	Wolf	♀	2.05.2009	38.21	S
Asali	Dog	♂	19.09.2010	34.01	S
Banzai	Dog	♂	2.04.2014	23.00	NS
Binti	Dog	♀	13.09.2010	25.00	S
Bora	Dog	♀	2.08.2011	20.57	NS
Enzi	Dog	♂	2.04.2014	29.01	S
Gombo	Dog	♂	21.03.2014	28.45	NS
Hiari	Dog	♂	21.03.2014	24.87	S
Imara	Dog	♀	21.03.2014	21.25	NS
Layla	Dog	♀	2.08.2011	21.45	S
Maisha	Dog	♂	18.12.2009	21.00	NS
Meru	Dog	♂	1.10.2010	34.30	S
Nia	Dog	♀	21.07.2011	17.00	NS
Panya	Dog	♀	2.04.2014	25.20	S
Sahibu	Dog	♂	21.03.2014	26.00	NS
Zuri	Dog	♀	24.05.2011	20.08	S

* Mean of weights in kg taken during the two tests

NS: non-social condition; S: social condition

### Experimental set-up

**Fig 1:**
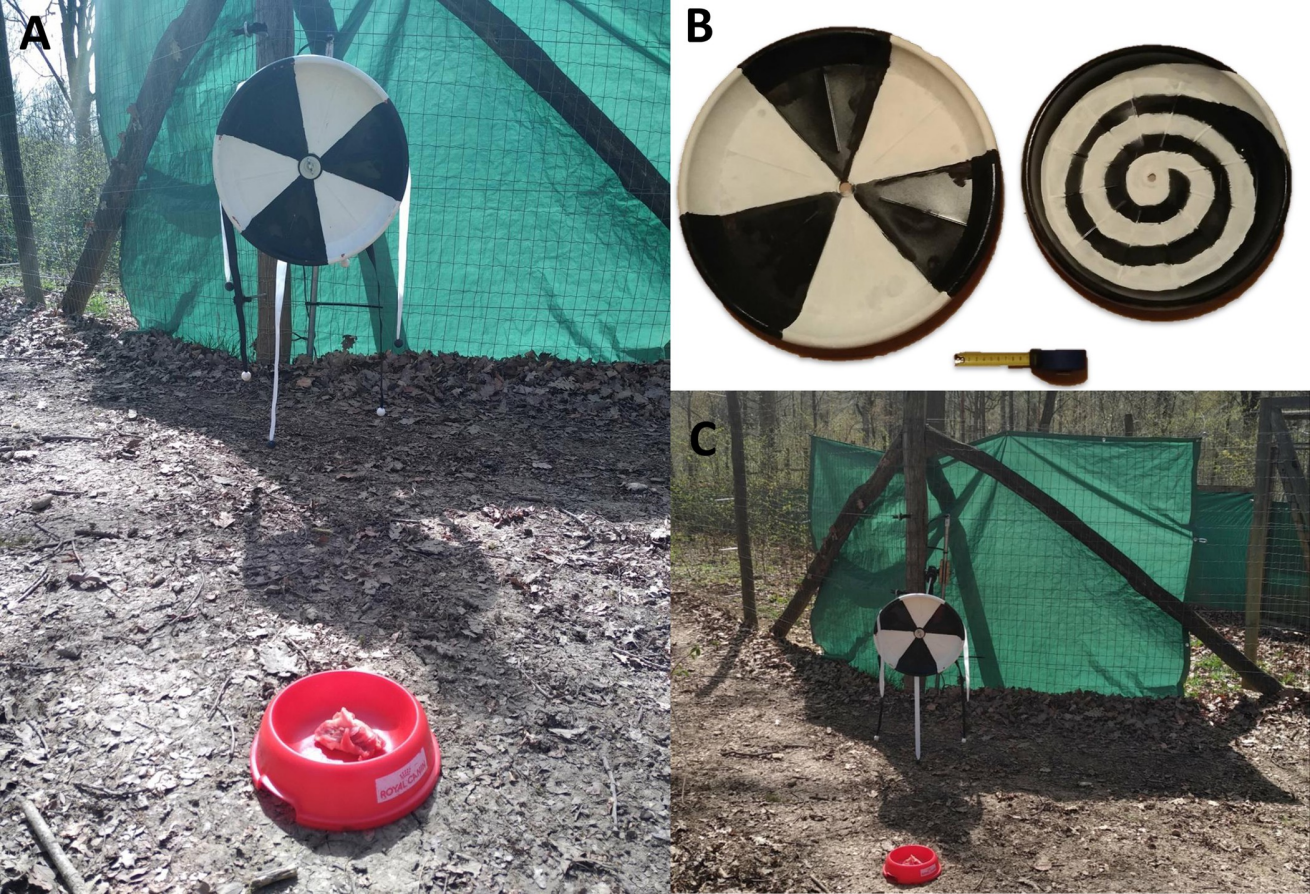
The experimental set-up we used. A) Close-up of the experimental set-up. B) The differently patterned discs used for the apparatus. C) Experimental set-up.

The experimental set-up was composed of a spinning object novel to the animals (i.e., the apparatus) and a bowl with food. The apparatus had three parts: (1) an ornated disc (i.e., ribbons with wooden marbles or ropes with plastic glasses), (2) a two-meter metal axis, and (3) a drill fixed on an 80 cm pole (see Fig 1). The drill was fixed outside the fence, then the metal axis was attached to the drill in order to go through the fence into the enclosure and the disk was attached to the other end of the axis. The drill acted as the motor of the apparatus. When turned on, the drill rotated the metal axis and the disc. The food was one piece of meat (≈ 100g)—a highly attractive reward for both wolves and dogs [79] -, placed 1 m in front of the spinning disc in a bowl well known by all the animals. Thus, the apparatus provided visual and acoustic stimulation via the gentle noise of the drill and the rotating ornaments.

Every subject was confronted with the experimental set-up (i.e., apparatus and food) once in each of two conditions: alone (non-social condition) or with a familiar human (social condition). The familiar human was defined as the trainer with the best relationship with the subject according to the judgment of the trainers themselves, a method which has been shown reliable [80]. Tests were separeted by at least one-month intervals between each condition. In addition, the size, pattern, and ornaments of the rotating disc differed between the two conditions to prevent habituation. The discs, sex, and age of the subjects were counterbalanced across conditions.

### Procedure

Each individual was tested in its home enclosure in the absence of its pack members. All the animals are shifted between enclosures of the Wolf Science Center on a regular basis and hence, are used to this procedure; packs are rotated between enclosures every few weeks so that each pack is familiar with each of the enclosures. Here we define home enclosure as the one where a pack had spent at least one night prior to the test. Before the beginning of each test session, the experimenters installed blinds on the fence of the home enclosure to ensure that neither the animal tested nor its pack members could see the installation of the experimental set-up (i.e., food and apparatus) or the actual testing. After these were installed, the entire pack was shifted out of the home enclosure. The subject was isolated from its pack and remained in the shifting system (i.e., a system of corridor-like enclosures used to move the animals between enclosures without direct contact with the trainer), whereas the rest of the pack was moved into the enclosure adjacent to the home enclosure. Meanwhile, an experimenter installed the experimental 15m ±2m away from the entrance, in its direct sight so it would be thing visible when entering the enclosure. The food was put in a bowl. Those bowls are used daily by the trainers to bring enrichment to the animals hence they associate the sight of the bowl with food. Once the subject was isolated from the pack, the trainers equipped the subject with a polar belt measuring cardiac outputs, a routine procedure for the animals. We used the Polar® RS800CX system (Polar Electro Oy, 2010) designed for human use [77, 81, 82]. It consists of three parts: a chest belt with electrodes, a clip-on to send measurements, and a watch-like data logger. First, an animal trainer wetted the belt with a mix of ethanol and water (70% ethanol to help the water wet the fur and 30% water, as it is better for signal transmission than ethanol) to improve conductivity, then the clip-on was fixed on the belt and the belt secured to the animal’s chest with the clip-on over the heart of the animal. Second, the trainer fastened the belt on the animal’s chest behind the shoulders and applied ethanol-water mix between the belt and the animal’s fur, again to enhance conductivity. The watch-like data logger was started and the quality of the signal between the clip-on and logger was checked. If the signal was suboptimal, the trainer adjusted the belt and the position of the clip-on or added more ethanol to the fur of the animal. Once the signal was satisfactory, the data logger was attached to an additional collar around the animal’s neck. After a two-minute waiting period (for the subject to calm down), the subject was released into its enclosure, the apparatus was turned on (i.e., the disc started to spin), and the test started. The test lasted until the animal ate all the food available or after a cutoff time of eight minutes in cases when the animal did not approach the food bowl. At the end of the test, the individual was shifted out of the enclosure again and after a two-minute waiting period (for the subject to calm down after the test), the HR device was taken off by a trainer. Afterward, the experimenter removed the experimental set-up from the enclosure, and the respective pack was shifted back to their home enclosure.

During the social condition, the familiar human entered the enclosure before the subject (i.e., while the subject was fitted with the belt and HR device) and stood roughly five body lengths of the respective animal tested away from the experimental set-up, on the right side, five metres for dogs and eight metres away for wolves to account for the differences in body size. The familiar human did not give food treats to the subject during the experiment. Additionally, during the test, the familiar human was instructed to act in a specific way, as described in Table 2 (see S1 Fig for more a schematic representation). The familiar human left the enclosure after the animal was shifted out of the enclosure at the end of the test. The trainer shifting and fastening the HR device was not the same person as the trainer acting as the familiar human in the social condition.

**Table 2:** Instructions for the human during the test.

Time (min)	Allowed Behaviours	Position
0 to 2	No interaction with the subject unless the subject initiates it^1^ No looking at the experimental setup No gaze alternation between experimental setup and subject	Stands immobile
2 to 4	Pointing at the food Gaze alternation between the subject and the food	Takes one step toward the experimental setup
4 to 6	Pointing at the food Gaze alternation between the subject and the food	Crosses half the distance towards the food
6 to 8	Call the subject’s name Gaze alternation (with clear head movement toward the food) Praising the subject in a cheerful voice Petting the subject if it seeks physical contact	Crouches down by the food

^1^ if the animal established eye contact, the familiar human would look back and, if approached, the person could talk to the animal in a cheerful but calm voice. If the animal touched the familiar human, she would crouch down and shortly pet the animal.

### Behavioural analysis

Each session was recorded with two cameras to capture the entire area surrounding the experimental set-up. The frequency, latency, and duration of several behaviours were coded with the Behavioral Observation Research Interactive Software (BORIS©; http://www.boris.unito.it/, [83]; see Table 3 for details). In addition, we coded what or whom (i.e., apparatus, food, familiar human) the wolves and the dogs chose to approach first. A choice was defined as coming within one body length of the apparatus, food, or familiar human while having the head and eyes fixed on either the apparatus, food, or familiar human. A naive observer coded independently 10% of the videos and then we calculated Inter-Observer Reliability (IOR). IOR was 82.7% of agreement.

**Table 3:** List of recorded behaviours (adapted from (24)).

Behaviours	Definition	Target	Type of coding
Avoidance	Being at the back of the enclosure, out of sight of the camera.		Duration
Close to entry/exit	Standing within one body length of the door/exit of the enclosure.		Duration, frequency
Jumping back	Brief movement backward, staring at the source of fear.		Frequency
Freeze	To stop moving and staring at the source of the fear.		Frequency
Vocalizations	To whine, whimper, growl, bark, or howl.		Frequency
Circling/Pacing	Walking or trotting back and forth.		Duration, frequency
Mouth liking	Tongue moved over the lips.		Frequency
Panting	To gasp for breath. The tongue is visibly moving inside and outside the mouth.		Frequency
Scratching	To nibble or scratch different body parts with front or hind paws.		Frequency
Shaking	To wiggle the whole body, starting with the head and finishing with the hind part of the body.		Frequency
Yawning	To open the mouth widely, slightly close the eyes and backward the ears.		Frequency
Approach	Moving forward within less than one body length.	Apparatus, familiar human, food	Frequency
Proximity	Staying within one body length of	Apparatus, familiar human, food	Duration, frequency

### Heart rate parameters analysis

We selected three sequences of HR for each individual, the first 30 seconds after the subject entered the enclosure (begin), the last 30 seconds before the subject took the food (end), and 30 seconds in between these two periods toward the middle of the HR recording (middle, the position of the middle period varied relatively to the time an individual took to reach the meat). The rationale for choosing these three sequences was that the beginning might reflect the first reaction to the experimental set-up whereas the middle could be reflective of the decision-making process, i.e, approaching or not approaching the experimental set-up, and the end would reflect the reaction of the animal when taking the food, or in the case they did not their physiological state at the arbitrary end of the test (i.e., after 8 minutes). The individuals with less than 30 seconds of recording were excluded from these analyses of the heart rate parameters (four individuals, three dogs, and one wolf, for both conditions and six individuals, three dogs and three wolves, for only one condition, each time these individuals approached and ate the food in less than 30 seconds). Individuals with recordings between 30 seconds and 1 minute had only two HR sequences (begin and end) to avoid using identical data points in the different HR sequences.

To sum up, all animals were recorded twice, once in the social condition and once in the non-social condition. In addition, the size, pattern, and ornaments of the rotating disc differed between the two conditions to prevent habituation. Those recordings range from 15 seconds to 8 minutes. Recordings shorter than 30 seconds were excluded from analyses and depending on the total duration of the test, we extracted two to three 30-second HR sequences from the whole HR recording. As the Polar system may produce artifacts [84–86], the resulting strings of raw data need to be edited and corrected, in this case using the algorithm-supported visual error correction (AVEC) of HR measurements [87]. Sequences with more than 5% errors were excluded from analyses. As a result, three individuals were completely excluded from the HR parameter analyses. The corrected data strings were then used to calculate one mean HR and one RMSSD (a proxy for the HRV, [8]) per 30-second strings with the software Kubios ©.

### Statistical analyses

We fitted the models in R (version 4.2.1; R Core Team, 2021) using the package lme4 (1.1–29; [88]) with the function lmer for the linear mixed model (LME, [89]) and function glmer for the generalized linear mixed model (GLMM; [89]). The package DHARMa (0.4.5; [90]) was used to test for overdispersion and zero-inflation, and the packages survival (3.2–10; [91]) and coxme (2.2–16; [92]) for the survival model used to analyzed latencies.

Several models did not converge during the analyses, and some did not reach statistical significance (see Table 4), to keep this manuscript concise, we choose to exclude them from the article’s main body but to present them in the supplementary materials.

**Table 4:** Summary of the variables analysed.

Variables tested	Statistical analyses	Results	Details in
1^st^ approach	Fisher test	Non-significant	Article
Final choice	Binomial GLMM	Significant	Article
Latency to take the food	Survival test	Significant	Article
Behaviours			
Proximity to food	LMM	Non-significant	Support. Info
Proximity to apparatus	LMM	No convergence	Support. InfoMaterial
Proximity to familiar human	LMM	No convergence	Support. Info
Circling	LMM	No convergence	Support. Info
Avoidance	LMM	No convergence	Support. Info
Proximity to entrance	LMM	No convergence	Support. Info
Nb approach to food	Poisson GLMM	Non-significant	Article
Nb approach to apparatus	Poisson GLMM	No convergence	Support. Info
Nb approach to familiar human	Poisson GLMM	No convergence	Support. Info
Stress- & fear-related behav.^1^	Negative binomial GLMM	Non-significant	Article
Cardiac output			
Heart Rate (HR; “mean” HR)	LMM	Significant	Article
Heart Rate Variability (HRV; RMSSD)	LMM	Non-significant	Article

^1^ Includes every occurrence of the following behaviours: avoidance, close to entry/exit, jumping back, freeze, Vocalizations, circling/pacing, Mouth liking, panting, scratching, shaking, yawning

GLMM: Generalized Linear Mixed Model; LMM: Linear Mixed Model. No convergence: During the analyses the models failed to converge therefore the analyses could not be completed.

#### 1. First approach

To better account for the low sample size, we used Fisher exact tests rather than Pearson Chi-square tests, to assess what or who dogs and wolves chose to approach first in each condition (social and non-social condition). Furthermore, to account for the increased risk of type I error caused by slitting the Data set in two by condition, we used Bonferroni correction to adjust the p-values [93].

The samples for the test were 23 data points for the social condition and 17 data points for the non-social condition. One dog and three wolves in the social condition, as well as seven dogs and four wolves in the non-social condition, refused to choose (i.e., did not come within one body length of the food, the apparatus, or the familiar human) and therefore were excluded from the analyses (see Appendix S3 Table in S1 File for a summary of each test sample size).

#### 2. Final choice

To analyze the proportion of dogs and wolves who successfully took the food, we use a binomial generalized linear mixed model (GLMM; [89]). The fixed factors were “species” (wolf or dog), condition (social or non-social), and their interaction. We added sex and age of the individual as control factors and identity of the animal was added to account for pseudo-replication as all animals were tested twice. To test the significance of the result, we compared the fit of the full model with that of a null model comprising only age, sex, and the random effect of individual using a likelihood ratio test [94]. We inspected Variance Inflation Factors (VIF, [95]) which we derived using the function VIF of the R-package car [96], applied to a standard linear model excluding the random effects and interactions, and found no collinearity issues. We checked for model stability by excluding subjects one at a time from the data and comparing the model estimates derived for these subsets of the data with those derived for the full data set. The interaction between “species” and conditions was unstable. Then, we compared the full model (“species” * conditions + age + sex + random factor animal identity) to its null model (sex+ age + random factor animal identity), using a likelihood ratio test (R function anova with argument test set to “Chisq”; [94]). To allow for a likelihood ratio test, we fitted the models using maximum likelihood (rather than Restricted Maximum Likelihood; [97]). P-values for the individual effects were based on likelihood ratio tests comparing the full of the respective reduced models ([98]; R function drop1).

The sample for the latency test was 56 data points.

#### 3. Latency to take the food

To understand whether latency to take the food differed depending on “species” (wolf or dog) and/or conditions (social or non-social) of the test, we fitted a survival model comprising the fixed factors “species”, conditions, and their interaction. Furthermore, sex and age of the individual, as well as, the order of the conditions, were included as control factors. Identity of the animal was added as a random factor to control for pseudo-replication. Then we compared this model, designated as the full model to a null model (comprising only sex, age, order of the condition, and the random factor animal identity).

#### 4. Behavioural responses

We also analyzed relevant behavioural responses. The continuous response variables such as proximity to the food were analyzed using separate linear mixed effect models (LME, [89]). The variable was fitted in a linear mixed model comprising of “species” (wolf or dog), condition (social or non-social), and their interaction as fixed factors. Sex, age of the individual, as well as the order of the conditions, were included as control factors. Subject identity was included as a random intercept to account for individual differences and to avoid pseudo-replication, as all subjects were tested in each condition. None of the random slopes and their correlations were identifiable; hence, we chose to not include them [98–100]. We then followed the method described above (section 2. Final choice, for detailed description see Appendix in S1 File).

The discrete response variables, i.e., number of approaches toward the food, and the number of stress- and fear-related behaviours were fitted using generalized linear mixed models using a poisson error structure. Each variable was fitted in a model comprising “species” (wolf or dog), condition (social or non-social), and their interaction as fixed factors. Sex and age of the individual as well as the order of the conditions were included as control factors, and identity of the animal was added as a random factor to control for pseudo-replication. These models were tested for overdispersion and zero-inflation with the package DHARMa. The model for the total number of stress and fear responses was heavily overdispersed (parameter dispersion: 3.44) and thus corrected using a negative binomial structure (package lme4, function glmer.nb).

The sample size for all the previous response variables above (discrete and continuous) was 55 data points collected on 28 animals tested twice each. One data point, i.e. Layla in the social condition, is missing due to an issue with the camera during the test.

#### 5. Cardiac outputs

To test whether cardiac output would differ between species depending on the conditions of the test the response variables “mean” HR and RMSSD (a proxy of the heart rate variability, HRV) were both analyzed in two separate linear mixed effect models (LME, [89]).“Species” (wolf or dog), condition of the test (social or non-social), order of the condition, and stage of the test (beginning, middle, or end) were included as fixed effects factors. We also included in the model the interaction between species and conditions to understand how wolves’ and dogs’ cardiac parameters were affected by the social environment. To control for the effects of temperature, body mass, age, sex, and success (i.e., the subject did take the food yes or no), these factors were also included as fixed effects. Subject identity was included as a random intercept to account for individual differences and to avoid pseudo-replication. None of the random slopes and their correlations were identifiable; hence, we chose to not include them [98–100]. We then followed the method described above (section 2. Final choice, for detailed description see Appendix S1 File).

The sample for the cardiac output models consisted of 75 data points after error corrections of the HR strands collected on 21 individuals.

## Results

### First approach

Three wolves and one dog never approached the food in any of the conditions, four wolves and three dogs approached the apparatus at least once. Nine dogs and three wolves approached the familiar human at least once (Appendix: S1 Table in S1 File).

**Fig 2:**
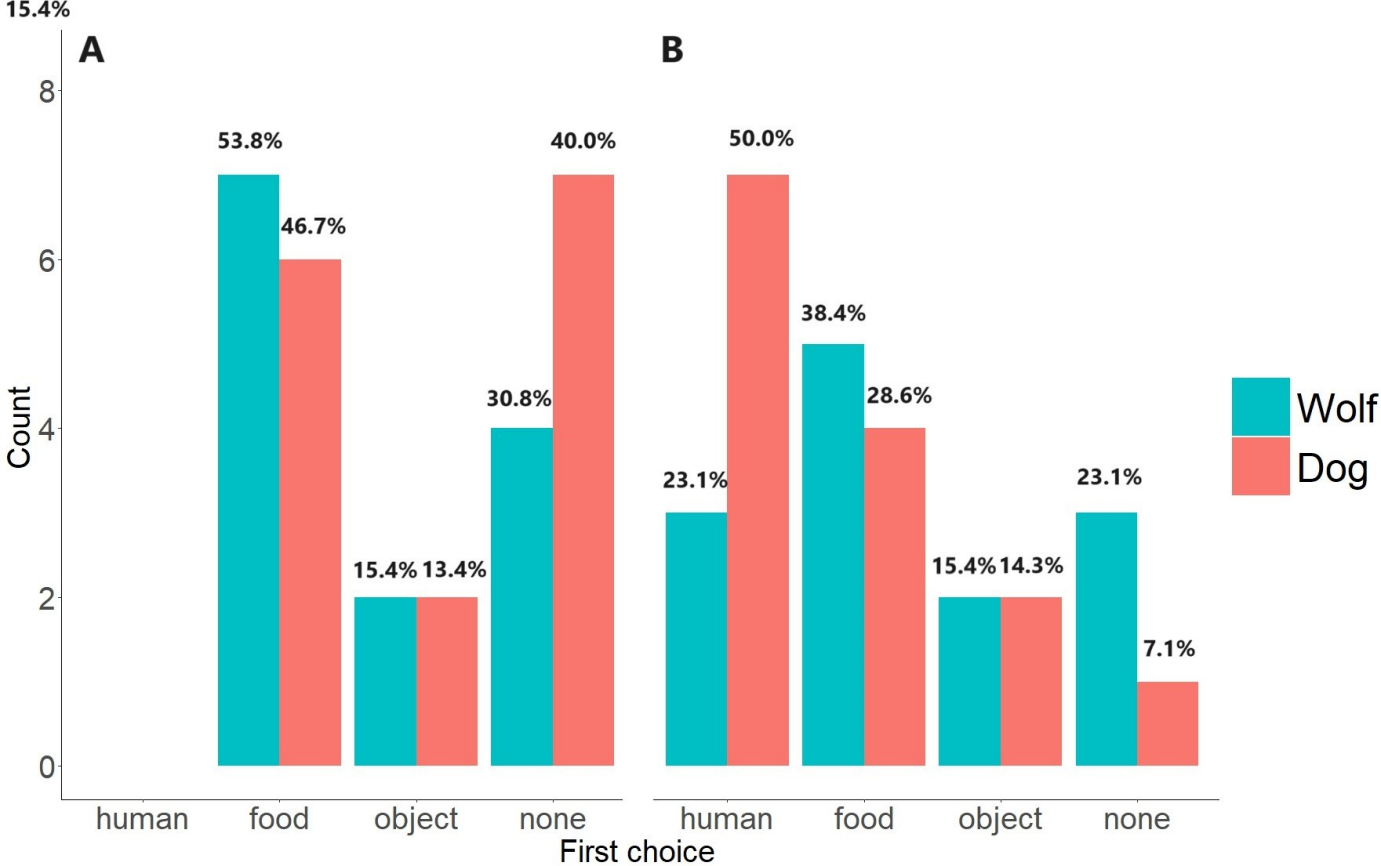
First approaches the wolves and dogs made after starting the test in A. non-social condition, B. social condition. Approach was defined as coming within one body length (head toward, eyes fixed on) towards either the apparatus (i.e., the object), the food, or the human.

During the non-social condition, animals neither showed a clear preference for the apparatus nor for the food (adjusted P>0.05 Fig 2A).

Also, the animals that approached the experimental setup during the social condition showed no clear preference for the apparatus, the food, or the familiar human (adjusted P>0.05; see Fig 2B).

### Final choice

Seven out of fifteen dogs took the food in non-social condition, whereas eight out of thirteen wolves did. In the social condition, fourteen out of fifteen dogs took the food whereas only seven out of thirteen wolves did. Overall, only one dog and five wolves never took the food in any of the conditions, whereas seven dogs and seven wolves always took the food.

**Fig 3:**
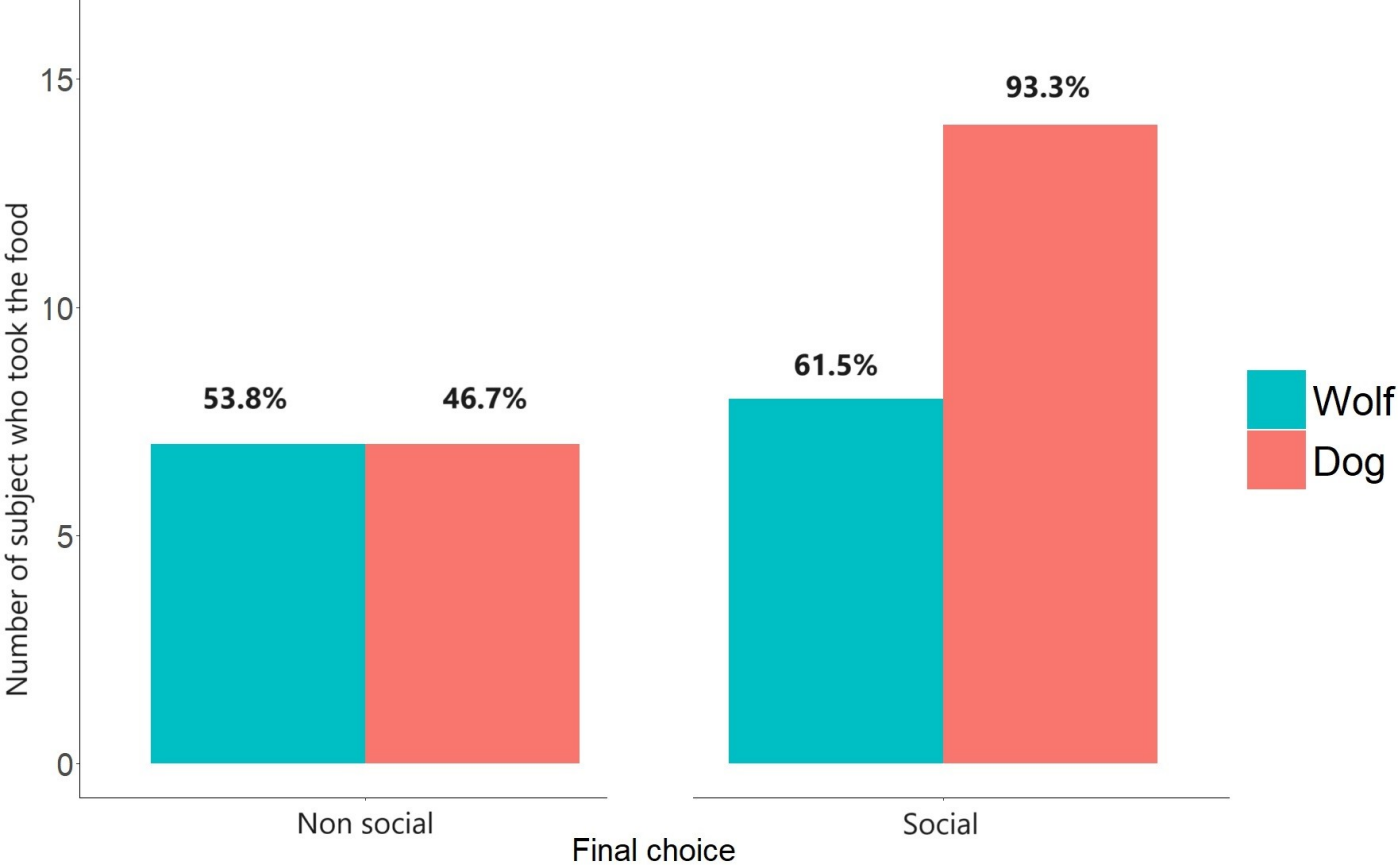
Number of wolves and dogs who made a positive final choice during the test by taking the food.

Overall, the full model (species * conditions + sex + age + animal ID) was statistically significant (likelihood ratio test: χ2 = 26.153, df = 2, P <0.005) compared to our null model (sex + age + animal ID). The interaction between “species” and conditions was non-significant, however as it was highly unstable, we removed it to explore the significance of “species” and conditions alone. Conditions were significant (χ2 = 25.028, df = 1, P <0.001, Fig 3, Table 5) but not “species” (χ2 = 1.403, df = 1, P >0.1). In the presence of a familiar human, both wolves and dogs were more likely to eat the food. Age was also significant (χ2 = 7.959, df = 1, P = 0.005, Table 5). Older animals were also more likely to eat the food.

**Table 5:** Results of the binomial GLMM model for the final choice.

	Estimate	SE	X^2^	df	P
(Intercept)	49.61	16.683			
Species (Wolf; Dog)	-12.209	6.865	1.403	1	0.2
Conditions (S; NS)	-21.737		25.028	1	<0.001
Sex (M; F)	0.79	4.294	7.959	1	0.8
Age	-6.786		0.034	1	0.005

### Latency to take the food

**Fig 4:**
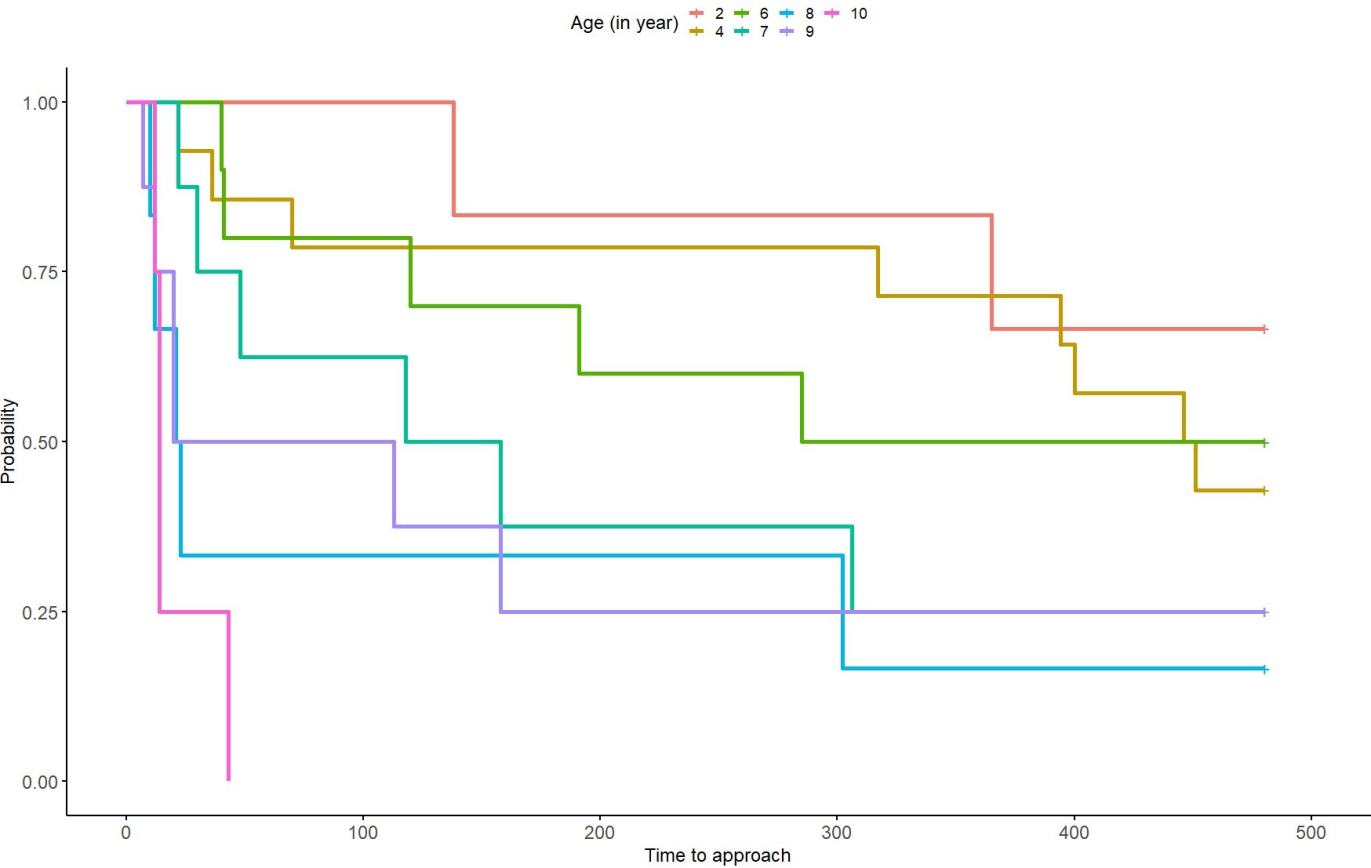
Latency to take the food in function of the age of the subjects. W = Wolves, D = Dog; Individuals per age group: 2 years old 3 W—0 D; 4 years old 0 W—7 D; 6 years old 5 W—0 D; 7 years old 0 W—4 D; 8 years old 0 W—3 D; 9 years old 3 W– 1 D; 10 years old 2 W– 0 D.

Overall, our full model (species * conditions + sex + age + order + animal ID) was statistically significant (likelihood ratio test: χ2 = 18.446, df = 6, P = 0.005) compared to our null model (animal ID). However, we found no effect of species, conditions, or their interaction on the latency to take the food. Age of the subject was the main factor affecting this latency (z = 3.42, P< 0.001; Fig 4, Table 6). The older the animals, the faster they were in taking the food.

**Table 6:** Results of the survival model for the latency to eat the food.

	Estimate	SE	Z	P
Species (Wolf; Dog)	-0.411	0.888	-0.46	0.640
Conditions (S; NS)	0.945	0.550	1.72	0.086
Sex (M; F))	0.595	0.755	0.79	0.430
Age	0.594	0.173	3.42	<0.001
Order (1; 2)	0.677	0.404	1.67	0.094
Species: Conditions	-0.930	0.815	-1.14	0.250

### Behavioural responses

We found no statistically significant difference in our wolves’ and dogs’ behaviours, be it the time spent in proximity of the food (χ2 = 4.54, df = 3, P = 0.2), in the number of approaches towards the food (χ2 = 7.49, df = 4, P = 0.11), or the number of stress-related and fear-related behaviours (χ2 = 9.70, df = 6, P = 0.13).

### Cardiac output models

**Fig 5:**
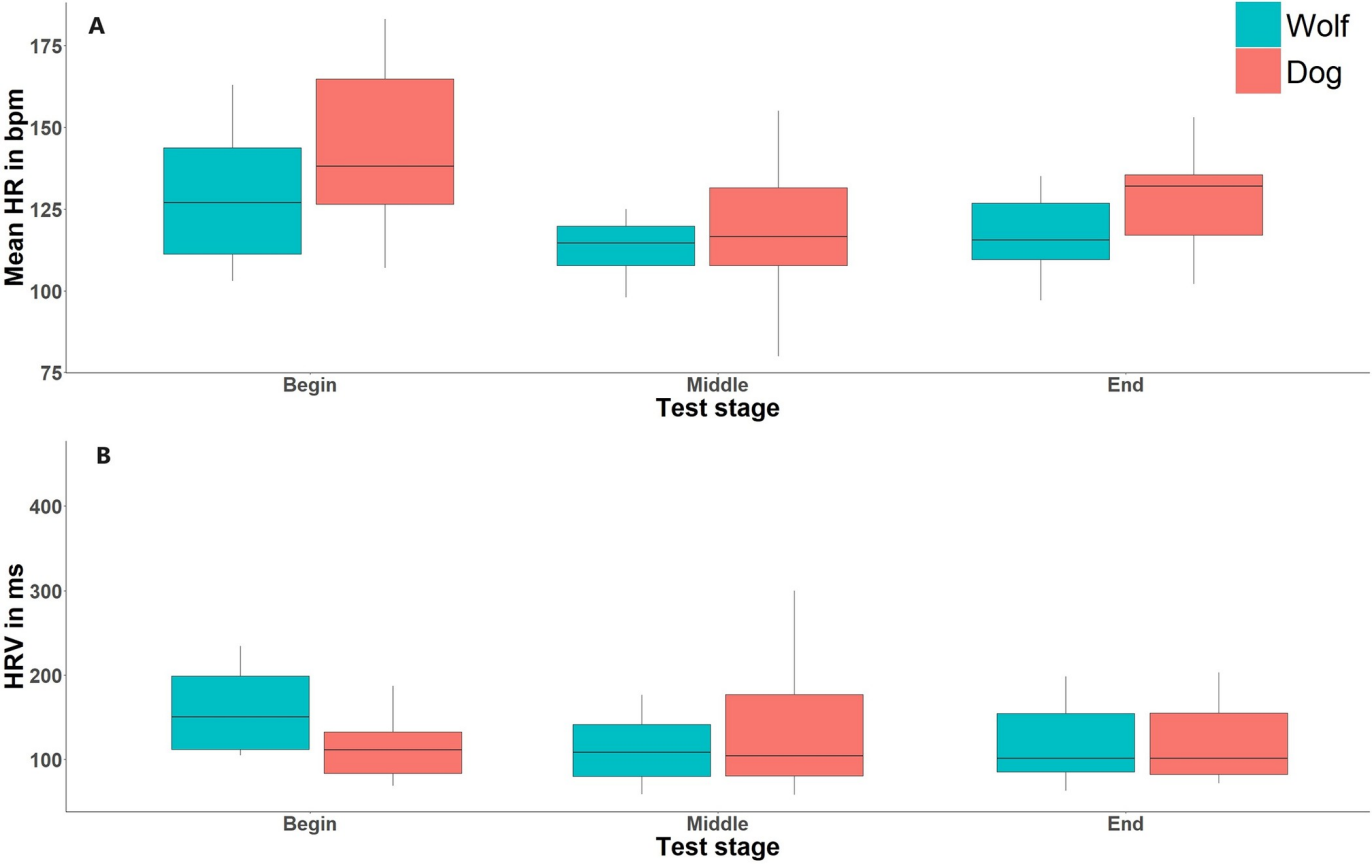
Boxplot of the cardiac output of the wolves and dogs. A) Mean HR in function of test stage. B) HRV as related to test stage. The whiskers represent the minimum (bottom) and maximum (top) data points, the edges of the box represent the interquartile (Q3–Q1) and the bold line is the median.

Our full HR model (species*conditions + temperature + order + sex + age + weight + test stage + success + animal ID) was statistically different from our null HR model (age + weight + temperature + sex + success + animal ID) suggesting that at least one of the factors of our full model affected our results (likelihood ratio test: χ2 = 18.56, df = 6, P< 0.005). Indeed, the stage of the test affected the mean HR of the animals: HR was higher at the beginning of the test when the animal first entered the enclosure. It then decreased during the test before increasing slightly at the end of the test, i.e., when most of the animals faced the apparatus to take the food (χ2 = 15.65, P<0.001, Fig 5A and Table 7 and Appendix: S2 Table in S1 File).

**Table 7:** Results of the heart rate model.

	Estimate	SE	X^2^	df	P
(Intercept)	163.114	21.406			
Species (Wolf; Dog)	-10.21	13.621			
Conditions (S; NS)	-1.185	6.325			
Temperature	-0.145	0.553	0.066	1	0.797
Order (1; 2)	-6.655	6.049	1.192	1	0.275
Age	-1.89	1.138	2.612	1	0.106
Sex (M; F)	-3.193	7.816	0.162	1	0.687
Weight	-0.209	0.803	0.067	1	0.796
Success (Yes; No)	2.325	6.68	0.120	1	0.729
HR Stage	-22.622	5.621	15.650	2	<0.001
	-14.495	4.751			
Species x Conditions	10.536	10.223	1.054	1	0.304

The comparison of our full HRV model against the null HVR model was not significant meaning that none of our factors of interest, i.e., species, condition, test stage, and their interaction influenced the RMSSD (likelihood test ratio: χ2 = 7.55, df = 6, P>0.05, Fig 5B).

## Discussion

In our risk-taking paradigm, equally raised and kept wolves and dogs behaved similarly: the older the wolf or dog, the faster it approached the food close to the spinning apparatus and the greater the probability of taking it. In addition, both were more likely to take the food in the presence of a familiar human than when alone, but this effect was greater in dogs. The main factor affecting cardiac parameters (mainly heart rate) was the stage of the test–start or end. Hence, our results support the *Canine Cooperation Hypothesis* as dogs and wolves demonstrated similar behaviour toward a familiar human in a risk-taking paradigm. Additionally, as wolves and dogs seemed to react similarly to human communicative gestures (two subjects, one wolf and one dog took the food after the 2 min time mark, when the familiar human starts pointing at the food; see Table 2), our results also support the *Two Stage Hypothesis*. However, they contrast with classic *Selection for Tameness Hypothesis* [18, 101–103] and the *Emotional Reactivity Hypothesi*s [62, 104] as dogs were not less reactive than wolves. Our results add to previous findings on dogs’ and wolves’ risk-proneness. WSC wolves and dogs were tested in a two-choice foraging paradigm before, with the result that wolves behaved more risk-prone than dogs [26]. In a novel object paradigm, wolves were found to be more neophobic than dogs but also more persistent [22]. The differences between the studies could be due to the paradigms used. The study by Marshall-Pescini et al. [26], involved a highly cognitive task that the dogs might have had difficulties understanding. Also, the latter study may have tested for risk proneness towards gains, whereas our paradigm rather tested for risk proneness towards loss (i.e. risk of injury as indicated by the unknown rotating object), this difference needs to be considered, as behavioural responses to potential risk are sensitive to context [105]. For comparing neophobia between wolves and dogs, Moretti et al. [22] presented a novel object to the subjects in their home enclosure and did not involve food; dogs seemed less interested than wolves, as half of them never even approached the objects. In the present study we combined a foraging context with a novel object. However, the novel object may not have elicited a clear fear response as the animals had already much experience with novel objects and moving apparatuses. Out of 15 dogs, only one did not approach the food in either condition—social or non-social. In contrast, three out of 13 wolves did not approach the food at all, including the two youngest individuals. As our main result was that mostly age affected the behavioural responses of the animals, this could also be the main reason for the differences between the three studies discussed above. In our study, the mean age was 6.4 years for wolves and 5.9 years for dogs. In contrast, the Marshall et al. study [26] reported mean ages of 4.7 years for wolves and 3.2 years for dogs. The Moretti et al. study [22] found mean ages of 1.7 years for wolves and 1.3 years for dogs. Hence, age and similar experiences in a rich environment (i.e., the animals at the WSC all frequently participate in different trainings and experiments) could have leveled out potential differences in the behavioural and physiological responses of wolves and dogs. This is supported by results indicating that the kind of functions dogs fulfill for their human partners (such as hunting, herding or guarding) affects their performance in experimental tests (discussed in [63]).

Also in other species, risk-proneness was shown to decrease with age [38, 68, 106–108]. Our results fit the life history theory framework, which holds that individuals balance their risk proneness with remaining lifetime reproductive odds. Hence, older individuals generally tend to be more risk-prone than younger ones (38). In fact, older wolves in the wild engage more in conflicts with other packs in defense of their kin than younger ones (49).

A surprising outcome of our study was that cardiac output was more affected by the stage of the test than by species, with the highest heart rates (HR) at the beginning, when the individual first entered the enclosure. This could be caused by expectancy arousal as generally, our animals are eager to participate in experiments. This was also found by Vasconcellos et al. (2016) in the form of enhanced salivary cortisol of wolves and dogs ahead of a training situation. Alternatively, individuals may have noticed the potential danger related to getting the desired food item, but this rather explains the second HR peak at the end of the test when the animals get close to the potentially dangerous object when approaching the food.

Vasconcellos et al. (2016) found that in an experimental one-to-one positive reinforcement training situation with wolves and dogs, while salivary cortisol decreased after a training session, wolves´ salivary cortisol was substantially lower than that of dogs before the training even started. This may reflect high inherent readiness for action in dogs in human-related tasks dogs as compared to wolves, manifest in their physiology. Alternatively, this may indicate that dogs’ physiology gears up in preparation for action with humans more than socialized wolves would. Unlike HR, we did not find any significant variation in heart rate variability (HRV) between wolves and dogs during our tests, although HRV tends to be higher in wolves than dogs, see Fig 5. This might be due to the short strings (30s) of cardiac output we analyzed. Indeed, other studies [77, 81, 82] had results with longer recordings (1 min 20s and 2 min). In our study, string length was constrained by study design, as we only analysed values until the animals ate the food.

The presence of the humans affected both dogs and wolves as they were both more likely to take the food in the presence of the familiar human. However, the effect was greater in dogs than in wolves. This is in agreement with previous studies where dogs did benefit from the social support of familiar people in potentially stressful situations [54, 60, 61] including a study at the WSC where, when separated from their pack, the WSC dogs displayed lower HR and higher HRV if they were resting near a familiar human, whereas most wolves did not. Hence, there was a greater calming effect due to the presence of a familiar human in dogs than the wolves [77].

As body mass and age [109–112] may affect cardiac output, we controlled for both parameters by adding them into the statistical models but found no influence of these parameters on HR and HRV in the full data set. Although in mammals, HR is generally negatively correlated with body mass [113], there is no clear evidence for this in dogs [114, 115]. In fact, some previous studies failed to show such a relationship [111, 116–118]. Moreover, studies that indicated a correlation between body mass and HR in dogs disagreed on its direction and the strength of this effect [112, 119, 120]. We are aware that the special situation of our subjects–highly socialized wolves and dogs kept in packs–does not allow to generalize our results to wild wolves or pet dogs. However, comparing wild wolves and pet dogs in an attempt to draw conclusions about domestication-related differences would be a futile exercise anyway, as life experience and socio-ecology of such wolves and dogs would differ widely. Therefore, we suggest that our results, unexpected as they are, are a valid contribution to the ongoing debate on domestication. Certainly, a bigger sample size would have been desirable. Alternatively, a more controlled, less naturalistic test design allowing for more repetition and fewer constraints on the HR strand lengths may reduce general noise levels, supporting statistical analysis.

To conclude, according to our paradigm, wolves’ and dogs’ risk-proneness varied with age, rather than species, and both dogs and our human-socialized socialized wolves seemed to respond behaviourally but hardly physiologically to the presence of a familiar human. This suggests that domestication effects may have been overshadowed by other factors such as age, life experience, and/or motivation. Generally, our results support previous suggestions that domestication should be investigated in a range of relevant contexts [77] rather than assuming that selection for tameness as the basic mechanism of domestication would necessarily produce robust predictions for all possible mechanisms, domains, and contexts. It seems that domestication does not uniformly affect all environmental responses and behaviours in dogs as compared to wolves but has rather produced a mosaic of context-dependent modifications [67, 121].

## Supporting information

S1 Fig Schematic representation of the different positions the familiar human holds during the test. (TIF)

S1 File Appendix. Contains S1 Table: Number of approaches; S2 Table: Descriptive statistics of the HR model; S3 Table: Final sample size per test; and the Detailed statistical Method.
(DOCX)

S2 File Dataset. Contains all the data sets used for the statistical analysis.
(XLSX)
