# Supplementary figures and images for: Decreased risk-proneness with increasing age in equally raised and kept wolves and dogs
Source: PLoS One. 2025 Jan 8;20(1):e0313916. doi: 10.1371/journal.pone.0313916 (PMC11709314; doi:10.1371/journal.pone.0313916)

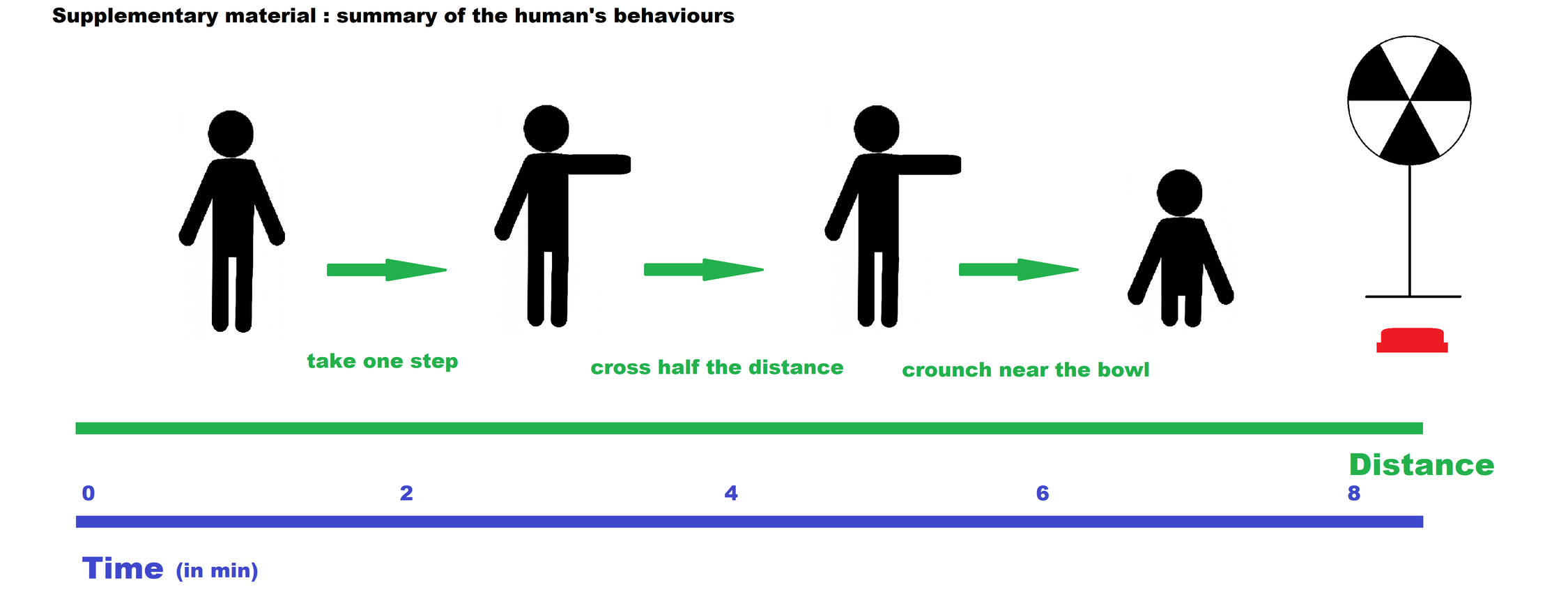

Supplement: S1 Fig — (TIF) [file pone.0313916.s001.tif]
