# Supplementary material for: Decreased risk-proneness with increasing age in equally raised and kept wolves and dogs
Source: PLoS One. 2025 Jan 8;20(1):e0313916. doi: 10.1371/journal.pone.0313916 (PMC11709314; doi:10.1371/journal.pone.0313916)
Supplement: S1 File — Contains S1 Table: Number of approaches; S2 Table: Descriptive statistics of the HR model; S3 Table: Final sample size per test; and the Detailed statistical Method. (DOCX) [file pone.0313916.s002.docx]

# APPENDIX

*Table S1: Number of approaches*

|  |  | Non-social condition | | Social condition | | Both | |
| --- | --- | --- | --- | --- | --- | --- | --- |
|  |  | Food | Apparatus | Food | Apparatus | Food | Apparatus |
| Never approached | Wolves | 4 | 11 | 3 | 11 | 3 | 9 |
|  | Dogs | 3 | 13 | 8 | 11 | 1 | 11 |
| Approached at least once | Wolves | 7 | 1 | 10 | 3 | 7 | 0 |
|  | Dogs | 6 | 2 | 13 | 3 | 6 | 1 |

Table S2: Descriptive statistics of the HR model

|  | Estimate | Std. Error | t value | X2.5. | X97.5. | min | max |
| --- | --- | --- | --- | --- | --- | --- | --- |
| (Intercept) | 163.114 | 21.406 | 7.62 | 119.859 | 205.076 | 151.016 | 173.717 |
| Species (Wolf; Dog) | -10.21 | 13.621 | -0.75 | -38.604 | 16.415 | -29.195 | -1.557 |
| Conditions (S; NS) | -1.185 | 6.325 | -0.187 | -13.281 | 10.957 | -4.287 | 3.75 |
| Temperature | -0.145 | 0.553 | -0.262 | -1.284 | 0.941 | -0.387 | 0.081 |
| Order (1; 2) | -6.655 | 6.049 | -1.1 | -18.45 | 5.463 | -9.565 | -4.355 |
| Age | -1.89 | 1.138 | -1.661 | -4.164 | 0.36 | -2.518 | -1.194 |
| Sex (M; F) | -3.193 | 7.816 | -0.409 | -18.881 | 12.084 | -12.709 | -0.213 |
| Weight | -0.209 | 0.803 | -0.26 | -1.726 | 1.421 | -0.623 | 0.79 |
| Success (Yes; No) | 2.325 | 6.68 | 0.348 | -10.135 | 15.27 | -3.969 | 6.064 |
| HR Stage | -22.622 | 5.621 | -4.025 | -33.467 | -12.175 | -26.069 | -19.117 |
|  | -14.495 | 4.751 | -3.051 | -23.586 | -5.233 | -16.835 | -11.052 |
| Species x Conditions | 10.536 | 10.223 | 1.031 | -9.01 | 30.736 | 4.529 | 16.721 |

Table S3: Final sample size per test

| Statistical Test | Total observed | | Excluded | | Total analysed | | |
| --- | --- | --- | --- | --- | --- | --- | --- |
|  | Dog | Wolf | Dog | Wolf | Dog | Wolf |  |
| Latency to take the food | 15 | 13 | 0 | 0 | 15 | 13 |  |
| First Choice  Social Condition | 15 | 13 | 1 | 3 | 14 | 10 |  |
| First Choice Non Social Condition | 15 | 13 | 4 | 7 | 11 | 6 |  |
| Final Choice | 15 | 13 | 0 | 0 | 15 | 13 |  |
| Behaviours | 15 | 13 | 1 | 0 | 14 | 13 |  |
| Cardiac Output | 15 | 13 | 4 | 3 | 11 | 10 |  |

**Detailed Statistical Method**

1. Behavioural variables

We also analyzed relevant behavioural responses. The continuous response variables such as proximity to the familiar human, the apparatus, or the food, time spent circling the experimental set-up (i.e. the food and the apparatus), time spent avoiding the experimental set-up, and time spent at the entrance of the enclosure were analyzed using separate linear mixed effect models (LME, (90)). Each variable was fitted in a linear mixed model comprising of “species” (wolf or dog), condition (social or non-social), and their interaction as fixed factors. Sex, age of the individual, as well as the order of the conditions, were included as control factors. Subject identity was included as a random intercept to account for individual differences and to avoid pseudo-replication, as all subjects were tested in each condition. None of the random slopes and their correlations were identifiable; hence, we chose to not include them (99–101). The models for the proximity to the familiar human and to the apparatus did not converge and therefore were excluded from further analyses. For each model that converged, we inspected visually the qqplot and the residuals plotted against the fitted values and found no obvious deviation from the normality and homogeneity assumptions. We checked for model stability by excluding subjects one at a time from the data and comparing the model estimates derived for these subsets of the data with those derived for the full data set. All the models were unstable for the interaction between species and conditions. We inspected VIF (96) and found no indication of collinearity. To avoid cryptic multiple testing and to keep type I error rate at the nominal level of 0.05 (102), we tested the significance of the full model as compared to the null model (comprising only age, sex, order of the conditions, and the random effects) through a likelihood ratio test (R function anova with argument test set to “Chisq”; (95)). To allow for a likelihood ratio test, we fitted the models using maximum likelihood (rather than Restricted Maximum Likelihood; (98)). P-values for the individual effects were based on likelihood ratio tests comparing the full of the respective reduced models ((99); R function drop1).

The discrete response variables, i.e., number of approaches toward the food, the familiar human, the apparatus, and the number of stress- and fear-related behaviours were fitted using generalized linear mixed models using a poisson error structure. Each variable was fitted in a model comprising “species” (wolf or dog), condition (social or non-social), and their interaction as fixed factors. Sex and age of the individual as well as the order of the conditions were included as control factors, and identity of the animal was added as a random factor to control for pseudo-replication. These models were tested for overdispersion and zero-inflation with the package DHARMa. The model for the total number of stress and fear responses was heavily overdispersed (parameter dispersion: 3.44) and thus corrected using a negative binomial structure (package lme4, function glmer.nb). Full models were then compared to their respective null models comprising only sex, age, order of the condition, and the random factor animal identity. Despite correction for overdispersion, the models for the number of approaches toward the apparatus and toward the humans did not converge and were therefore excluded from further analyses.

The sample size for all the previous response variables above (discrete and continuous) was 55 data points collected on 28 animals tested twice each. One data point, i.e. Layla in the social condition, is missing due to an issue with the camera during the test.

1. Cardiac outputs

To test whether cardiac output would differ between species depending on the conditions of the test the response variables “mean” HR and RMSSD (a proxi of the heart rate variability, HRV) were both analyzed in two separate linear mixed effect models (LME, (90)).“Species” (wolf or dog), condition of the test (social or non-social), order of the condition, and stage of the test (beginning, middle, or end) were included as fixed effects factors. We also included in the model the interaction between species and conditions to understand how wolves’ and dogs’ cardiac parameters were affected by the social environment. To control for the effects of temperature, body mass, age, sex, and success (i.e., the subject did take the food yes or no), these factors were also included as fixed effects. Subject identity was included as a random intercept to account for individual differences and to avoid pseudo-replication. None of the random slopes and their correlations were identifiable; hence, we chose to not include them (97–99). Visual inspection of the qqplot and the residuals plotted against the fitted values indicated no obvious deviation from the normality and homogeneity assumptions. We used the method described above to check model stability and found the models stable. VIF inspection (94) revealed that species and body mass were collinear with a VIF of 8.12 and 9.80, respectively. However, there was also a considerable variation of body mass within both wolves and dogs; hence, the results obtained for these two predictors should not be distorted by collinearity among them. We tested the significance of the full model as compared to the null model (comprising only age, body mass, sex, temperature, and the random effects) using a likelihood ratio test (R function anova with argument test set to “Chisq”; (93)). To allow for a likelihood ratio test, we fitted the models using maximum likelihood (rather than Restricted Maximum Likelihood; (96)). P-values for the individual effects were based on likelihood ratio tests comparing the full of the respective reduced models ((97); R function drop1).

The sample for the cardiac outputs models consisted of 75 data points after error corrections of the HR strands collected on 21 individuals.
